# Clinical application of genetics to guide prevention and treatment of oral diseases

**DOI:** 10.1111/cge.12396

**Published:** 2014-01-01

**Authors:** KS Kornman, PJ Polverini

**Affiliations:** a Department of R&D, Interleukin GeneticsWaltham, MA, USA,; b Department of Pathology, University of Michigan School of DentistryAnn Arbor, MI, USA

**Keywords:** caries, genetics, oral cancer, periodontitis

## Abstract

Dental care costs in the United States exceed $100 billion annually. Personalized medicine efforts in dentistry are driven by potentially compelling clinical utility and cost-effectiveness prospects in the major diseases of periodontitis, caries, and oral cancers. This review discusses progress and challenges identifying genetic markers and showing clinical utility in dentistry. Genome-wide association studies (GWAS) of chronic periodontitis (CP) identified no significant variants, but CDKN2BAS variants on chromosome 9 were significantly associated with aggressive periodontitis. Stratifying patients by interleukin (IL)-1 gene variants, smoking and diabetes differentiated CP prevention outcomes. Dental caries’ GWAS identified significant signals in LYZL2, AJAp1, and KPNA4; and efforts are ongoing to identify genetic factors for multiple caries phenotypes. Trials of molecularly targeted therapies are in progress for oral, head, and neck squamous cell carcinomas (OHNSCC) and results have been promising but limited in their effectiveness. Current opportunities and challenges for molecular targeting for OHNSCC are discussed.

## Conflict of interest

K. S. K. is a fulltime employee, officer, and shareholder of Interleukin Genetics, Inc., which has patents on the use of genetic variations to manage various diseases, including periodontitis, which represents a potential conflict relative to this manuscript. P. J. P. declares no conflicts of interest relative to this manuscript.

Dental care is a substantial cost to healthcare, involving 500 million office visits annually and exceeding $100 billion 1. The primary diseases in terms of demand for care and costs are dental caries and periodontal disease, and new oral cancers are estimated to exceed 500,000 annually 2 with substantial morbidity and mortality. Personalized medicine as proposed in dentistry involves the use of genetic information after an initial clinical diagnosis to guide treatment decisions for oral cancers, moderate to severe periodontitis, and severe caries, to better manage differential responses to standard therapies. Genetic information also may be used to guide more aggressive prevention protocols for severe chronic periodontitis (CP) and severe caries. This narrative review provides perspective on current evidence supporting use of genetics to stratify patients for improved prevention and treatment of major oral diseases. Details of the search strategies for periodontitis and dental caries and uses of the relevant papers identified to inform the overviews are provided in Appendix S1, Supporting Information.

## Periodontitis

### Chronic periodontitis

CP, a bacterially-induced chronic inflammatory disease, destroys bone and connective tissues supporting teeth. CP affects 47% of United States adults with 8.5% having severe disease 3. Moderate/severe periodontitis leads to tooth loss; elevated inflammatory mediators 4; and risk for other diseases 5. Adult dental prophylaxis, primarily for CP prevention, is among the most widely used healthcare services 6.

Bacteria initiate periodontitis, but genetic and environmental factors influence CP severity 7, with approximately 50% of severity variance attributable to genetics 8.

Practical challenges complicate the study of CP genetics:

1. Few data sets have sufficient subjects with both periodontitis and genetic parameters.
2. Periodontitis classification systems, developed for epidemiology or treatment needs assessments are not ideal for genetic exposure studies. For example, extraction of severely diseased teeth may reduce patients' severity classifications and exclude cases completely if protocols require minimum teeth numbers.

Systematic reviews limited to a few candidate genes have addressed association with CP. Two genome-wide association studies (GWAS) have been reported 9,10. Both used well-defined case criteria, adjusted for confounders, and were modestly powered [4504 9; 3365 10]. Neither found variants with GW significance. Six loci with suggestive evidence of association in Divaris et al. 9 were not associated in Teumer et al. 10. In Divaris 9, additive effects of all genome-wide SNPs explained 18% of severe periodontitis heritable variance, which increased to 52% when smoking interactions were considered. Moreover, novel pathways were tagged and warrant further exploration. In Teumer et al. 10, additive effects of risk alleles together explained 34% of disease variance, supporting concepts that complex traits are cumulative result of alleles with weak effects.

Approximately 38 disease-associated markers have been reported from CP candidate gene studies, most of which were underpowered and not replicated. A small subset of variants are supported by meta-analyses, including in IL1A, IL1B, IL1RN, IL6, IL10, FcγR, TLR4, and MMP1 genes, but most studies were underpowered which may propagate type I errors. Some adequately powered studies support CP association for IL1 variants 11,12 but another 13 did not support association for any genes implicated in meta-analyses. IL-1 gene variations are the most commonly studied associations with severe/progressive CP, due in part to early reports of association 14. Functional IL1 variants have allele-specific differences in transcription factor binding 15, white cell IL-1β expression 16, and gingival fluid IL-1β levels (*n* = 900) 17. Because elevated IL-1β levels are implicated in CP progression 18, including primate model evidence that IL-1 blocking drugs reduce periodontitis 19, the role of IL1 variants in severe CP has biological plausibility.

## Personalized medicine opportunities in CP

Evidence suggests opportunities for patient stratification using genetic plus non-genetic information to improve prevention/management of severe generalized CP, which occurs in 8–15% of adults 3. Of patients treated for CP, disease progression continues in 20–25% 20, and limited evidence supports twice yearly preventive care for adults without periodontitis 21. Uses for diagnosis of a symptomatic patient or prediction of CP are not envisioned.

No randomized controlled trials have assessed genetic influences on different preventive/treatment approaches, but multiple studies have longitudinally monitored post-treatment outcomes and retrospectively evaluated influences of smoking, diabetes and limited genetic variants. Clinical impact of these risk factors is probably actionable by more rigorous bacterial control and certain anti-inflammatory agents.

To date, clinical utility studies of CP genetics have involved primarily IL1 gene variations together with smoking and diabetes. Using a claims database, 5117 adults with no periodontitis history were stratified by pre-defined criteria, and those negative for three risk factors (smoking, diabetes, IL-1 genotype) were not less likely to lose teeth over 16 years with two cleanings/year compared to one (p = 0.092), but patients with ≥1 risk factor benefited from two cleanings/year compared to one (p = 0.002) 22. This is consistent with a 10-year prevention study in which tooth loss was associated with smoking and IL-1 genotype 23. Genetic influence on long-term outcomes of treated CP patients is less clear 24–26. Contrary to prediction of complex diseases, use of genetic information as in the above CP prevention study 22, has shown strong clinical value in discriminating responses to prevention/treatment of some complex diseases 27,28.

Pre-requisites for clinically useful CP genetic factors include: markers validated in adequately powered studies involving severe/progressive CP, consideration of non-genetic risk factors, i.e. smoking and diabetes; evidence of allele-specific biological effects with plausible role in CP; and association of pre-defined risk strata, including genetic and non-genetic factors, with long-term studies of CP severity/progression or impact on prevention/treatment outcomes.

IL-1 genes were not identified in the CP GWAS. Possible explanations are: (i) prior IL-1 associations with severe CP were false, (ii) IL-1 risk-associated genotypes include multiple promoter haplotypes not tagged by any single SNP, as generally used in GWAS, and (iii) IL-1 gene effects are dependent on gene–environment interactions. Some challenges to CP genetic studies are characteristic of complex diseases. The discovery/validation phase requires well-defined phenotypes, and markers in LD with causal variants often vary across populations thereby complicating validation. Other challenges are specific to periodontitis. Bacterial exposure alone is sufficient for CP initiation, but long-term bacterial exposure measurements are rarely available. Because risk factors, including genetics, influence CP severity/progression, some genetic effects may be undetectable in populations with low bacterial exposure.

### Aggressive periodontitis

Aggressive periodontitis (AgP) refers to uncommon forms of bacterially-induced periodontitis not associated with known systemic conditions or genetic syndromes that substantially modify susceptibility and/or progression of disease 29. AgP is generally responsive to anti-microbial therapies but differ from CP with earlier onset age, more rapid progression, and often less clinical inflammation and bacterial mass. Prevalence of AgP is less than 1% 30 but is enriched in certain populations 31.

An AgP GWAS found GWA with an intronic SNP in the glucosyltransferase gene *GLT6D1*, for which the associated risk allele showed substantially reduced binding of transcription factor GATA3 in cell models 32. The non-coding RNA ANRIL (CDKN2BAS; chr.9p21.3), is among best replicated risk genes for atherosclerosis. Multiple SNPs in this region have been validated for AgP association, making ANRIL the best replicated AgP risk gene to date 13,33–35. CDKN2BAS markers associated with CP in a Dutch population were not replicated in a larger German sample after multiple testing correction 35. Type II errors are difficult to exclude in such studies because of limited sample sizes and strong age and life-style influences on this phenotype.

Specific isoforms of ANRIL have been implicated in regulation of fatty acid and glucose metabolism 34 pathways that may influence pathogenesis. *COX2* 36 and *IL10* genes 13, also have been associated with risk for AgP in sufficiently powered case–control studies.

AgP is a heterogeneous genetic disorder with environmental interactions. Currently known risk alleles have a relatively high frequency that precludes use for population screening, because few who test positive will develop this uncommon disease. Once AgP is diagnosed, genetic information may help stratify patients by different etiologies to guide therapy, but no current evidence supports that use.

### Dental caries

Dental caries, a common chronic disease, results from specific tooth-adherent microbial biofilms that demineralize tooth structure by metabolizing dietary sugars to produce acid 37. Fermentable carbohydrates enrich cariogenic bacteria, including *Streptococus mutans*, *S. sobrinus*, and *Lactobacillus* species, in the biofilm leading to dental decalcification. Severe-early childhood caries (S-ECC), affecting multiple smooth tooth surfaces before age 5, can lead to pain, abscess formation, and loss of teeth. Although all age cohorts experience dental caries, children represent the primary health concern. S-ECC is associated with more new carious lesions 38 and emergency room visits, increased treatment costs 39, delayed development 40, and diminished ability to learn 41. S-ECC prevalence varies by socio-economic status, with one kindergarten group exhibiting a 9.5% prevalence with 5.69 mean affected teeth 42.

Although environmental factors, including dietary composition, access to fluoride and dental care, and oral hygiene practices influence S-ECC, host factors including salivary composition, enamel structure, taste preferences, and immune responses vary among children and may be genetically determined 43. Childhood caries has strong heritability, with strongest effect in primary dentitions 44,45. Inconsistent associations have been reported for childhood caries and genetic variants involved in enamel/dentin mineralization, salivary composition, and matrix metalloproteinases 46–48.

Of two GWAS of permanent dentition caries, one found two significant loci, LYZL2 which involves anti-bacterial defenses, and AJAP1 which may influence tooth development, and the other found no significant associations but both studies identified several novel loci with non-significant associations 46,49. No associations overlapped in the two studies. Two childhood caries GWAS have been reported. One found no variants with significant associations, and suggestive associations did not replicate in independent populations 50, and the other found significant association between KPNA4 and replicated the association with AJAP1 51.

Although one may envision risk stratification for S-ECC at diagnosis of first smooth surface lesions to guide intervention opportunities, investigators have appropriately questioned the clinical utility of genetic information in management of at-risk populations.

## Personalized oral and head and neck oncology

Personalized cancer therapy has proven to be an effective strategy for more than a decade 52,53. As genomic technology and genetic profiling advance identification of gene expression patterns, new phenotypic details will facilitate accurate matching of patient needs with precision-based therapies 54,55.

Approximately 500,000 new cases of oral and head and neck squamous cell carcinoma (OHNSCC), are expected to arise this year 2,56. Many of these patients will present with advanced stage disease at the time of diagnosis. Despite improvements in therapy, strategies designed to improve early diagnosis and minimize disease progression have remained elusive 57,58.

Many systematic reviews have assessed the association between specific candidate genes and risk for OHNSCC 59,60. This review is focused on the role of genomics in guiding therapy for OHNSCC and will not address genetic markers associated with presence of OHNSCC in general.

Ongoing discovery efforts have revealed a wide range of potential targets for tumor therapy. Examples include among others, the tyrosine kinase inhibitor, imatinib (Gleevec), in the treatment of chronic myelogenous leukemia, Herceptin in breast cancer therapy and the B-RAF kinase inhibitor PLX4032 in the treatment of melanoma 61–63. Despite rapid advances in ‘omics’ technology, the pace of progress in linking targeted therapies with well-characterized patient profiling has been slow to develop for OHNSCC 64,65.

Targeted therapies for the treatment of squamous cell carcinoma are being evaluated in a number of clinical trials 64–70. These targets include among others, oncogenes, biomarkers associated with epithelial–mesenchymal transition, gene amplifications, gene mutation, translocations and signaling pathways that regulate cell growth, cell motility and survival 71. Some of the more promising targets include the epidermal growth factor receptor (EGFR), vascular endothelial growth factor (VEGF) and the intercellular signaling pathways MAPK/Erk and phosphatidylinositol-3′ kinase (PI3)/Akt/mammalian target of rapamycin (mTOR) 72–76. The efficacy of current target-specific agents are greatly enhanced and side effects are significantly reduced when used in combination with chemoradiation 66–69. This duel-targeting approach has proven to be successful for the treatment of human papillomavirus (HPV)-positive cancers of the head and neck 77. However, results to date with targeted therapies for the treatment of non-HPV-positive OHNSCC have had mixed results 72. Despite these setbacks, current and emerging genomics-based targeting strategies hold great promise.

One of the earliest identified targets for the treatment of patients with OHNSCC was the tyrosine kinase receptor EGFR 78,79. Upon binding to EGF and tumor necrosis factor alpha (TNF-α), signaling pathways are activated with important downstream effects on tumor growth, mobility, survival and therapeutic resistance 78,79. Whereas monoclonal antibodies to EGFR, such as cetuximab, panitumab, nimotuzumab, and zalutumumab and tyrosine kinase inhibitors have been shown to disrupt downstream signaling they have limited clinical utility when used as single agents 67–69,80. In contrast, when used in combination with conventional radiotherapy or chemotherapy significant improvement in loco-regional control has been reported 67–69. Although EGFR is over expressed in the majority of HNSCC, only a small subset of tumors has shown clinical responsiveness to EGFR therapy 73,81. Because there are no biomarkers that predict clinical responsiveness the search for new targets with improved clinical efficacy continues.

Another class of inhibitors that has received considerable attention are the PI3-Akt/mTOR pathway inhibitors 58,74,75. Mutations in PI3ck and PTEN oncogenes that activate the mTOR pathway are a feature of most human malignancies 82,83 and has been shown to play a central role in tumor progression and therapeutic resistance 75. Inhibition of mTOR by rapamycin and less toxic derivative inhibitors have promising antitumor activity when used alone or in combination with conventional chemotherapy agents in clinical trials 75,76,84.

Angiogenesis inhibitors have long been proposed as an effective therapeutic target for a wide variety of tumors 85–87. The principal anti-angiogenic targets have been introduced above, including VEGF and downstream components of the signaling pathway that regulate VEGF-mediated processes including angiogenesis, cell survival and therapeutic resistance. As tumors adapt to chronic stress, molecular chaperones associated with the unfolded protein response can activate the angiogenic switch and confer therapeutic resistance to tumors 88. For example, bevacizumab, a humanized VEGF monoclonal antibody, inhibits angiogenesis and facilitates the delivery of chemotherapeutic agents by increasing vascular permeability 87. However, the effectiveness of VEGF/VEGFR agents such as bevacizumab, sunitinib or sorafenib when used as single agents or in combination with chemoradiation has been limited and in some cases their use discontinued because of life threatening side effects 89–91.

As investigations into the genomics and unique molecular architecture of cancers continue new therapeutic targets will no doubt be revealed. Biomarkers present in saliva have already revealed a number of new genetic and epigenetic targets 92,93. New targeting agents such as broad spectrum kinase inhibitors show great promise in clinical trials 94,95. Other potential targeting agents include proteasome inhibitors, histone deacetylases, heat shock proteins and other molecular chaperones 58,72. Innovative approaches to therapy such as oncolytic viruses and systemic immunotherapy among others may prove to be a value in the near future 96. While many challenges lie ahead new biomarkers will no doubt provide further insight into how to therapeutically navigate and target with precision the molecular networks and genetic mutations that drive neoplastic development and progression.

## Supporting Information

The following Supporting information is available for this article:

Appendix S1. Literature search criteria and supplemental references.
