# Supplementary material for: Clinical application of genetics to guide prevention and treatment of oral diseases
Source: Clin Genet. 2014 Jan 1;86(1):44–9. doi: 10.1111/cge.12396 (PMC4233973; doi:10.1111/cge.12396)
Supplement: Supplementary file 1 — Supplementary [file cge0086-0044-SD1.doc]

# Supplemental material

**Publication Search Strategy**

This narrative review started with a comprehensive search strategy to identify original research publications involving genetic variations associated with chronic periodontitis, aggressive periodontitis, and dental caries. Details of the search strategy and uses of the relevant papers identified are provided in Supplemental Material. All papers meeting selection criteria were reviewed for their contributions to the general summaries developed for each of the topics.

Detailed search strategies were developed for PubMed (National Center for Biotechnology Information at the U.S. National Library of Medicine) and EMBASE (Excerpta Medica Database from Elsevier Life Science Solutions). Databases were searched for papers published from January 1, 1997 through November 30, 2013 using MeSH (Medical Subject Headings) terms and key words, and Boolean operators (OR, AND) were used to combine terms and searches. Only studies on humans and only publications in the English language were included. Three searches (shown below) were conducted for genetics of periodontitis and publications were stratified into those with a “chronic periodontitis” or an “aggressive periodontitis” phenotype, as described by the authors. Some papers evaluated both phenotypes. The abstracts of all papers were reviewed for relevance to the topic. The periodontitis searches produced 184 relevant papers on chronic periodontitis and 90 relevant papers on aggressive periodontitis. One search (shown below) was conducted for genetics of dental caries and produced 22 relevant papers.

For the sections on chronic periodontitis, aggressive periodontitis, and dental caries, we decided to focus the reviews on genome-wide association studies (GWAS) and genes with supporting meta-analyses. All relevant papers were then reviewed for additional perspectives which should be included to appropriately represent the work in the area, given the focus on clinical application and the limitations of manuscript length.

**Periodontitis Searches**

"chronic periodontitis"[All Fields] AND ("polymorphism, genetic"[MeSH Terms] OR ("polymorphism"[All Fields] AND "genetic"[All Fields]) OR "genetic polymorphism"[All Fields] OR ("genetic"[All Fields] AND "polymorphism"[All Fields])) AND (("1997/01/01"[PDAT] : "2013/11/30"[PDAT]) AND "humans"[MeSH Terms])

"periodontitis"[All Fields] AND ("polymorphism, genetic"[MeSH Terms] OR ("polymorphism"[All Fields] AND "genetic"[All Fields]) OR "genetic polymorphism"[All Fields] OR ("genetic"[All Fields] AND "polymorphism"[All Fields])) AND (("1997/01/01"[PDAT] : "2013/11/30"[PDAT]) AND "humans"[MeSH Terms])

"aggressive periodontitis"[All Fields] AND ("polymorphism, genetic"[MeSH Terms] OR ("polymorphism"[All Fields] AND "genetic"[All Fields]) OR "genetic polymorphism"[All Fields] OR ("genetic"[All Fields] AND "polymorphism"[All Fields])) AND (("1997/01/01"[PDAT] : "2013/11/30"[PDAT]) AND "humans"[MeSH Terms])

**Dental Caries Search**

"dental caries"[All Fields] AND ("polymorphism, genetic"[MeSH Terms] OR ("polymorphism"[All Fields] AND "genetic"[All Fields]) OR "genetic polymorphism"[All Fields] OR ("genetic"[All Fields] AND "polymorphism"[All Fields])) AND (("1997/01/01"[PDAT] : "2013/11/30"[PDAT]) AND "humans"[MeSH Terms])

**Supplemental References**

Ang KK, Andratschke NH, Milas L. Epidermal growth factor receptor and response of head-and-neck carcinoma to therapy. *International journal of radiation oncology, biology, physics* 2004;58:959-965.

Armitage GC. Development of a classification system for periodontal diseases and conditions. *Ann Periodontol* 1999;4:1-6.

Bourhis J, Rivera F, Mesia R, et al. Phase I/II study of cetuximab in combination with cisplatin or carboplatin and fluorouracil in patients with recurrent or metastatic squamous cell carcinoma of the head and neck. *J Clin Oncol* 2006;24:2866-2872.

Chin L, Andersen JN, Futreal PA. Cancer genomics: from discovery science to personalized medicine. *Nat Med* 2011;17:297-303.

Choong NW, Cohen EE. Epidermal growth factor receptor directed therapy in head and neck cancer. *Critical reviews in oncology/hematology* 2006;57:25-43.

Dietrich T, Sharma P, Walter C, Weston P, Beck J. The epidemiological evidence behind the association between periodontitis and incident atherosclerotic cardiovascular disease. Journal of periodontology 2013;84:S70-84.

Ehmke B, Kress W, Karch H, Grimm T, Klaiber B, Flemmig TF. Interleukin-1 haplotype and periodontal disease progression following therapy. *Journal of clinical periodontology* 1999;26:810-813.

Elser C, Siu LL, Winquist E, et al. Phase II trial of sorafenib in patients with recurrent or metastatic squamous cell carcinoma of the head and neck or nasopharyngeal carcinoma. *J Clin Oncol* 2007;25:3766-3773.

Frederick BA, Helfrich BA, Coldren CD, et al. Epithelial to mesenchymal transition predicts gefitinib resistance in cell lines of head and neck squamous cell carcinoma and non-small cell lung carcinoma. *Molecular cancer therapeutics* 2007;6:1683-1691.

Harari PM, Huang S. Radiation combined with EGFR signal inhibitors: head and neck cancer focus. *Seminars in radiation oncology* 2006;16:38-44.

Hart TC, Atkinson JC. Mendelian forms of periodontitis. *Periodontology 2000* 2007;45:95-112.

Konig J, Ruhling A, Plagmann HC, Meisel P, Kocher T. Influence of interleukin (IL)-1 composite genotype on clinical variables in non-smoking, well-maintained compliant patients with chronic periodontitis. *Swed Dent J* 2005;29:11-16.

Lang NP, Tonetti MS, Suter J, Sorrell J, Duff GW, Kornman KS. Effect of interleukin-1 gene polymorphisms on gingival inflammation assessed by bleeding on probing in a periodontal maintenance population. *J Periodontal Res* 2000;35:102-107.

Li D, Cai Q, Ma L, et al. Association between MMP-1 g.-1607dupG polymorphism and periodontitis susceptibility: a meta-analysis. *PloS one* 2013;8:e59513.

Morgillo F, Bareschino MA, Bianco R, Tortora G, Ciardiello F. Primary and acquired resistance to anti-EGFR targeted drugs in cancer therapy. *Differentiation; research in biological diversity* 2007;75:788-799.

Offit K. Personalized medicine: new genomics, old lessons. *Hum Genet* 2011;130:3-14.

Persson GR, Matuliene G, Ramseier CA, Persson RE, Tonetti MS, Lang NP. Influence of interleukin-1 gene polymorphism on the outcome of supportive periodontal therapy explored by a multi-factorial periodontal risk assessment model (PRA). *Oral Health Prev Dent* 2003;1:17-27.

Pretzl B, Wiedemann D, Cosgarea R, et al. Effort and costs of tooth preservation in supportive periodontal treatment in a German population. *J Clin Periodontol* 2009;36:669-676.

Rodemann HP, Dittmann K, Toulany M. Radiation-induced EGFR-signaling and control of DNA-damage repair. *International journal of radiation biology* 2007;83:781-791.

Ross JS. Cancer biomarkers, companion diagnostics and personalized oncology. *Biomarkers in medicine* 2011;5:277-279.

Siegel R, Naishadham D, Jemal A. Cancer statistics, 2013. *CA: a cancer journal for clinicians* 2013;63:11 30.

Song GG, Choi SJ, Ji JD, Lee YH. Association between tumor necrosis factor-alpha promoter -308 A/G, -238 A/G, interleukin-6 -174 G/C and -572 G/C polymorphisms and periodontal disease: a meta-analysis. *Molecular biology reports* 2013;40:5191-5203.

Teoh DC, Rodger S, Say J, Hartley A. Hypofractionated radiotherapy plus cetuximab in locally advanced head and neck cancer. *Clinical oncology* 2008;20:717-720.

Zheng J, Gao L, Hou T, et al. Association between TLR4 polymorphism and periodontitis susceptibility: a meta-analysis. *Crit Rev Eukaryot Gene Expr* 2013;23:257-264.
